# Anxiety and prognosis of patients with myocardial infarction: A meta‐analysis

**DOI:** 10.1002/clc.23605

**Published:** 2021-05-07

**Authors:** Yi Wen, Yuan Yang, Jian Shen, Suxin Luo

**Affiliations:** ^1^ Department of Cardiology The First Affiliated Hospital of Chongqing Medical University Chongqing China

**Keywords:** anxiety, meta‐analysis, myocardial infarction, prognosis

## Abstract

Although anxiety is highly prevalent after myocardial infarction (MI), but the association between anxiety and MI is not well established. This study aimed to provide an updated and comprehensive evaluation of the association between anxiety and short‐term and long‐term prognoses in patients with MI. Anxiety is associated with poor short‐term and long‐term prognoses in patients with MI. We performed a systematic search in the PubMed and Cochrane databases (January 2000–October 2020). The study endpoints were complications, all‐cause mortality, cardiac mortality, and/or major adverse cardiac events (MACEs). Pooled data were synthesized using Stata SE12.0 and expressed as risk ratios (RRs) and 95% confidence intervals (CIs). We included 9373 patients with MI from 16 published studies. Pooled analyses indicated a correlation between high anxiety and poor clinical outcomes (RR: 1.19, 95% CI: 1.13–1.26, *p* < .001), poor short‐term complications (RR: 1.23, 95% CI: 1.09–1.38, *p* = .001), and poor long‐term prognosis (RR: 1.27, 95% CI: 1.13–1.44, *p* < .001). Anxiety was also specifically associated with long‐term mortality (RR: 1.16, 95% CI: 1.01–1.33, *p* = .033) and long‐term MACEs (RR: 1.54, 95% CI: 1.26–1.90, *p* < .001). This study provided strong evidence that increased anxiety was associated with poor prognosis in patients with MI. Further analysis revealed that MI patients with anxiety had a 23% increased risk of short‐term complications and a 27% increased risk of adverse long‐term prognosis compared to those without anxiety.

## 1 INTRODUCTION

Cardiovascular disease, especially ischemic heart disease (IHD), is the leading cause of death and disability globally.^1^ Myocardial infarction (MI) is a common IHD with a high morbidity and mortality. Although reperfusion interventions have improved mortality associated with MI, acute MI (AMI) is a life‐threatening disease worldwide.^2^, ^3^ Many factors affect the outcomes of MI, such as age, sex, prior MI,^4^ hypertension, smoking, dyslipidemia, diabetes,^5^ and prior stroke.^6^ In recent years, numerous studies have shown that emotional distress, especially depression and anxiety, plays an adverse role in the prognosis of MI.^7^, ^8^

Depression is a risk factor for adverse medical outcomes in patients with acute coronary syndrome (ACS).^9^ Depression after AMI is associated with fatal and non‐fatal events, adverse health status outcomes, and increased costs.^10^, ^11^, ^12^, ^13^ Anxiety is also highly prevalent after MI in approximately 50% patients.^14^ However, the association between anxiety and MI is not well established. Some studies have reported that anxiety is a risk factor for poor MI prognosis,^14^, ^15^, ^16^, ^17^, ^18^, ^19^, ^20^, ^21^, ^22^ but other studies did not find any such association.^23^, ^24^, ^25^, ^26^, ^27^, ^28^, ^29^

We aimed to discover the relationship between anxiety and short‐term and long‐term prognoses in patients with MI.

## 2 METHODS

### 2.1 Aim

This study was a meta‐analysis of prospective studies. We sought to compare the clinical outcomes between MI patients with and without anxiety. We included all studies available from January 2000 to October 2020. Systematic identification, appraisal, synthesis, statistical aggregation, and reporting of results were performed in accordance with known guidelines.^30^, ^31^

### 2.2 Literature search

A systematic search was performed in the PubMed and Cochrane databases using keyword‐based queries with the following terms: “myocardial infarction” and “anxiety.” The results were limited to human studies published in English. Moreover, reference lists of original and review articles were manually screened as a supplement. This meta‐analysis was performed according to the Preferred Reporting Items for Systematic Reviews and Meta‐Analyses (PRISMA) statement.^32^

### 2.3 Selection

Studies were identified by two independent raters, and any disagreement between them was resolved via consensus. Articles meeting the following criteria were included: (1) prospective studies that enrolled patients with established AMI; (2) AMI patients diagnosed with anxiety using reliable and validated instruments; (3) studies including clinical outcomes in patients and documenting the risk ratios (RRs) and their corresponding 95% confidence intervals (CIs) for clinical outcomes in patients with anxiety compared with those in patients without anxiety.

### 2.4 End points

The prognosis of this meta‐analysis focused on the short‐term complications and long‐term mortality or adverse events. The study endpoints were complications, all‐cause mortality, cardiac mortality, and/or major adverse cardiac events (MACEs). Complications include recurrent ischemia, or reinfarction, or ventricular arrhythmia, or congestive heart failure (CHF), or death during hospitalization. MACEs include cardiac death, reinfarction, readmission for recurrent ischemia, and ventricular arrhythmia. Recurrent ischemia was defiend as new onset of chest pain, ST‐segment changes on the electrocardiogram, or hemodynamic instability. Reinfarction was evidenced by recurrent positive creatine kinase‐MB. Ventricular arrhythmia was defined as ventricular tachycardia lasting more than 15 s, or any ventricular tachycardia requiring pharmacological and/or electrical intervention due to hemodynamic instability or chest pain, or ventricular fibrillation. CHF was identified either through a diagnosis in the discharge summary, or both (a) chest X‐ray consistent with CHF and (b) acute administration of diuretic or other agent to treat CHF.

### 2.5 Statistical analysis

The pooled RR and its corresponding 95% CI for prognosis were calculated using Stata SE12.0 (StataCorp., College Station, Texas). The I^2^ test and Q statistic test were used to assess the heterogeneity among the included studies. When there was no obvious heterogeneity, pooled data were analyzed using a fixed‐effects model; otherwise, the random‐effects model was used (*p* < .1 and/or I^2^ > 50% suggested significant heterogeneity). Publication bias was evaluated by visual inspection of the funnel plots. Sensitivity and subgroup analyses were performed to evaluate the stability of the results. The “adjusting for depression” was defined as depression was included in the adjustment factors of including studies. A *p*‐value of <.05 was considered statistically significant.

## 3 RESULTS

**FIGURE 1:**
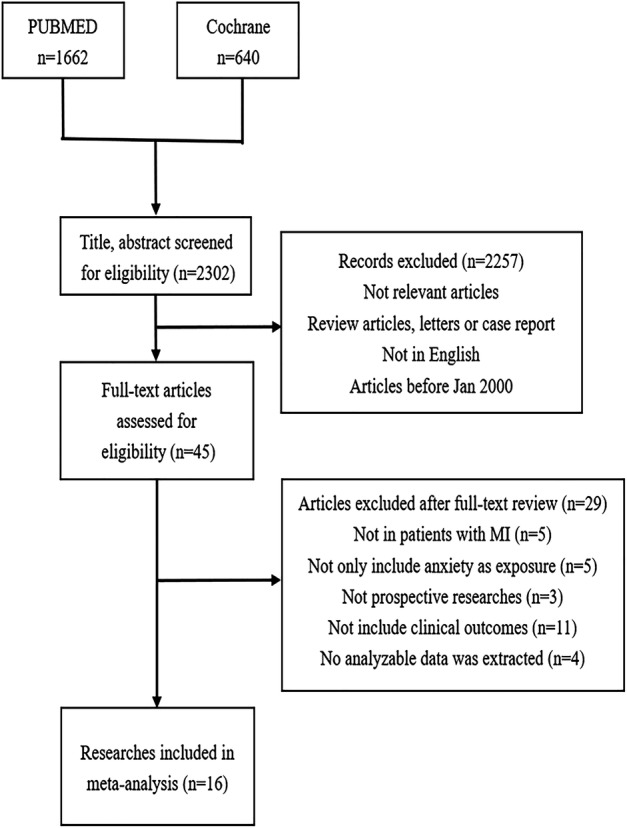
Flow chart of searching results. CI, confidence interval; MI, myocardial infarction; RR, risk ratio

A flowchart of the search results is shown in Figure 1. A total of 2302 studies were initially searched. After screening the title, abstract, and full text, 16 studies were finally included in this meta‐analysis.^14^, ^15^, ^16^, ^17^, ^18^, ^19^, ^20^, ^21^, ^22^, ^23^, ^24^, ^25^, ^26^, ^27^, ^28^, ^29^

### 3.1 Study characteristics

The main information reported in the 16 included studies is presented in Table 1. A total of 9373 patients with MI were included in this meta‐analysis. Their mean age ranged from 58 to 67 years, and 61.2%–100% patients were men. Various valid instruments were used to evaluate anxiety. Two studies assessed anxiety at a median of 4 days post‐MI and was interviewed to obtain information about the immediate experiences during 0–2 h prior to MI onset^15^ and more distant experiences in the 24–26 h prior to MI^26^ by trained research staff; the other 14 studies evaluated anxiety after MI.^14^, ^16^, ^17^, ^18^, ^19^, ^20^, ^21^, ^22^, ^23^, ^24^, ^25^, ^27^, ^28^, ^29^ In these studies, approximately 5.5%–58.2% patients with MI presented with anxiety. Short‐term follow‐up was defined as a follow‐up duration of <1‐year, including four studies,^17^, ^19^, ^20^, ^21^ and long‐term follow up was defined as a follow‐up duration of ≥1‐year, including 12 studies.^14^, ^15^, ^16^, ^18^, ^22^, ^23^, ^24^, ^25^, ^26^, ^27^, ^28^, ^29^ Four studies reported a significant association between anxiety and short‐term prognosis.^17^, ^19^, ^20^, ^21^ Five studies reported a significant association between anxiety and long‐term prognosis (1 on all‐cause mortality, 4 on MACEs)^14^, ^15^, ^16^, ^18^, ^22^; the other seven studies did not report a significant association between anxiety and long‐term prognosis (3 on all‐cause mortality, 5 on cardiac mortality, 2 on MACEs).^23^, ^24^, ^25^, ^26^, ^27^, ^28^, ^29^ The result of RR was adjusted called multivariable RR, including 13 studies.^14^, ^15^, ^16^, ^17^, ^18^, ^19^, ^20^, ^21^, ^23^, ^24^, ^25^, ^26^, ^27^

**TABLE 1:** Design and characteristics of included researches

Study	Country	Patients	(*n*)	Mean age (year)	Male (%)	Instrument	Anxiety (%)	Anxiety assessment time	Follow‐up	End points	Adjustment
Smeijers, 2017^15^	United States	MI	2176	60.1	70.8	STPI	9.4	0–2 h prior to MI	10	All‐cause mortality	Age, gender, race/ethnicity, marital status, education, income, smoking status, alcohol consumption, BMI, usual physical activity, medical history of MI, HTN, DM and CV medications
Van Beek, 2016^16^	The Netherlands	MI	193	62.1	65.7	CAQ	NA	2–7 days after admission	4.2	MACE^a^	Age, gender, LVEF, history of MI and BDI score
Iles‐Smith, 2015^17^	United Kingdom	STEMI	202	59.6	75.7	HADS‐A	NA	<14 days after discharge	0.5	Complications^b^	SAQ angina frequency, SAQ angina stability, the GRACE score and the CCI
Larsen, 2014^23^	Denmark	MI	896	67.0	69.2	HADS‐A	23.6	12–14 weeks after discharge	2.6	All‐cause mortality + MACE^c^	Age, gender, smoking, previous MI, HTN, DM, CV medication, physical activity, and depression
Hosseini, 2014^24^	Iran	MI	285	59.1	69.1	STAI	50.9	2–15 days after MI	5	Cardiac mortality	Age, gender, smoking, alcohol consumption, previous MI, HTN, DM, thrombolysis therapy and depression
Roest, 2014^25^	The Netherlands	MI	418	59.0	81.1	HARS	9.8	2 months after MI	3.8	MACE^d^	Age, gender, cardiac history, and LVEF
Wrenn, 2013^26^	United States	MI	1944	60.2	69.2	STPI	9.2	24–26 h prior to MI	10	All‐cause mortality + cardiac mortality	Age, sex, BMI, marital status, race, educational attainment, smoking, previous MI, CHF, DM, HTN, noncardiac comorbidities, CV medications, social status, and alcohol consumption
Roest, 2012^18^	The Netherlands	MI	438	61.0	80.8	CIDI	5.5	3 months after MI	5.7	MACE^e^	Age, gender, LVEF
AbuRuz, 2011^19^	United States	MI	322	61.0	61.2	BSI	49.4	72 h after admission	in‐hospital	Complications^f^	Marital status, age, sex, history of DM, HTN, MI, use of anxiolytic agents, smoking history, peak level of chest pain, SBP and DBP at admission, Killip classification at admission, and daily β‐blocker dose
Huffman, 2008^20^	United States	MI	110	62.7	78.0	BAI	29	72 h after admission	in‐hospital	Complications^g^	Gender, history of DM, HTN, BDI‐II scores, current MDD, LVEF and peak troponin T
Moser, 2007^21^	Multi‐center	MI	536	62.0	66.0	BSI	48.9	72 h after admission	in‐hospital	Complications^h^	Age, sex, HTN, DM, previous MI, LVEF, type of MI, peak pain level, admission Killip class, aspirin administration in the emergency department, beta‐blocker dministration in the emergency department, anxiolytic given during hospitalization
Benninghoven, 2006^22^	Germany	MI	76	NA	80.0	STAI	30.3	<7 days after MI	1.3	MACE^i^	not adjustment
Strik, 2003^14^	The Netherlands	MI	318	58.0	100	SCL‐90	58.2	1 month after MI	3.4	MACE^j^	Age, LVEF ≤50%, depression, hostility, use of antidepressants
Frasure‐Smith, 2003^27^	Canada	MI	896	59.4	74.1	STAI	13.4	During hospital admission	5	Cardiac mortality	Age, gender, educational level, daily smoking, previous MI, thrombolytic treatment at index admission, Q‐wave MI, Killip class>1, revascularization at index
Lane, 2002^28^	United Kingdom	MI	288	62.7	75	STAI	26.1	2–15 days after MI	3	Cardiac mortality	Not adjustment
Welin, 2000^29^	Sweden	MI	275	NA	83.6	STAI	41.8	3–6 days after MI	10	Cardiac mortality + all‐cause mortality	Not adjustment

Abbreviations: BAI, Beck Anxiety Inventory; BDI, Beck Depression Inventory; BMI, body mass index; BSI, Brief Symptom Inventory; CAQ, Cardiac Anxiety Questionnaire; CCI, Charlson Co‐morbidity Index; CHF, chronic heart failure; CIDI, Composite International Diagnostic Interview; CV, cardiovascular; DBP, diastolic blood pressure; DM, diabetes mellitus; GRACE, Global Registry of Acute Coronary Events; HADS‐A, Hospital Anxiety and Depression Scale—Anxiety subscale; HARS, Hamilton Anxiety Rating Scale; HTN, hypertension; LVEF, left ventricular ejection fraction; MDD, major depressive disorder; MI, myocardial infarction; NA, not available; SAQ, Seattle Angina Questionnaire; SBP, systolic blood pressure; SCL‐90, 90‐item Symptom Check List; STAI, State–Trait Anxiety Inventory; STPI, State–Trait Personality Inventory.

^a^ Cardiac mortality or a readmission for ischemia, ventricular flutter, ventricular fibrillation, heart attack, CABG/PCI treatment.

^b^ Readmission for potential ischemic heart disease.

^c^ MI, heart failure, stroke, or transient ischemic attack.

^d^ Cardiac mortality and/or recurrent MI.

^e^ Cardiac mortality or ischemic heart disease, cardiac arrhythmia, heart failure, cerebrovascular disease, peripheral vascular disease.

^f^ Acute recurrent ischemia, reinfarction, ventricular tachycardia, ventricular fibrillation, in‐hospital death.

^g^ Recurrent chest pain, recurrent chest pain with ischemia, ventricular arrhythmia, ventricular arrhythmia requiring intervention, congestive heart failure, recurrent MI.

^h^ Acute recurrent ischemia, reinfarction, ventricular tachycardia, ventricular fibrillation, in‐hospital death.

^i^ Cardiovascular death, reinfarction and revascularization.

^j^ Cardiac death or recurrent MI.

### 3.2 Quality assessment

The methodological quality of eligible studies was evaluated using the Newcastle‐Ottawa quality assessment scale (NOS).^33^ The methodological quality of the studies were judged using three main criteria: the selection of the study groups, comparability of the groups, and ascertainment of either the exposure or outcome of interest for studies. The total NOS score was ranged from 1 to 9. Studies with NOS scores ≥6 were classified as high‐ or medium‐quality studies, while studies with NOS scores <6 were classified as low‐quality studies. The quality assessment of the 16 included studies is shown in Suppl Table 1.

### 3.3 Anxiety and clinical outcomes in patients with MI

**FIGURE 2:**
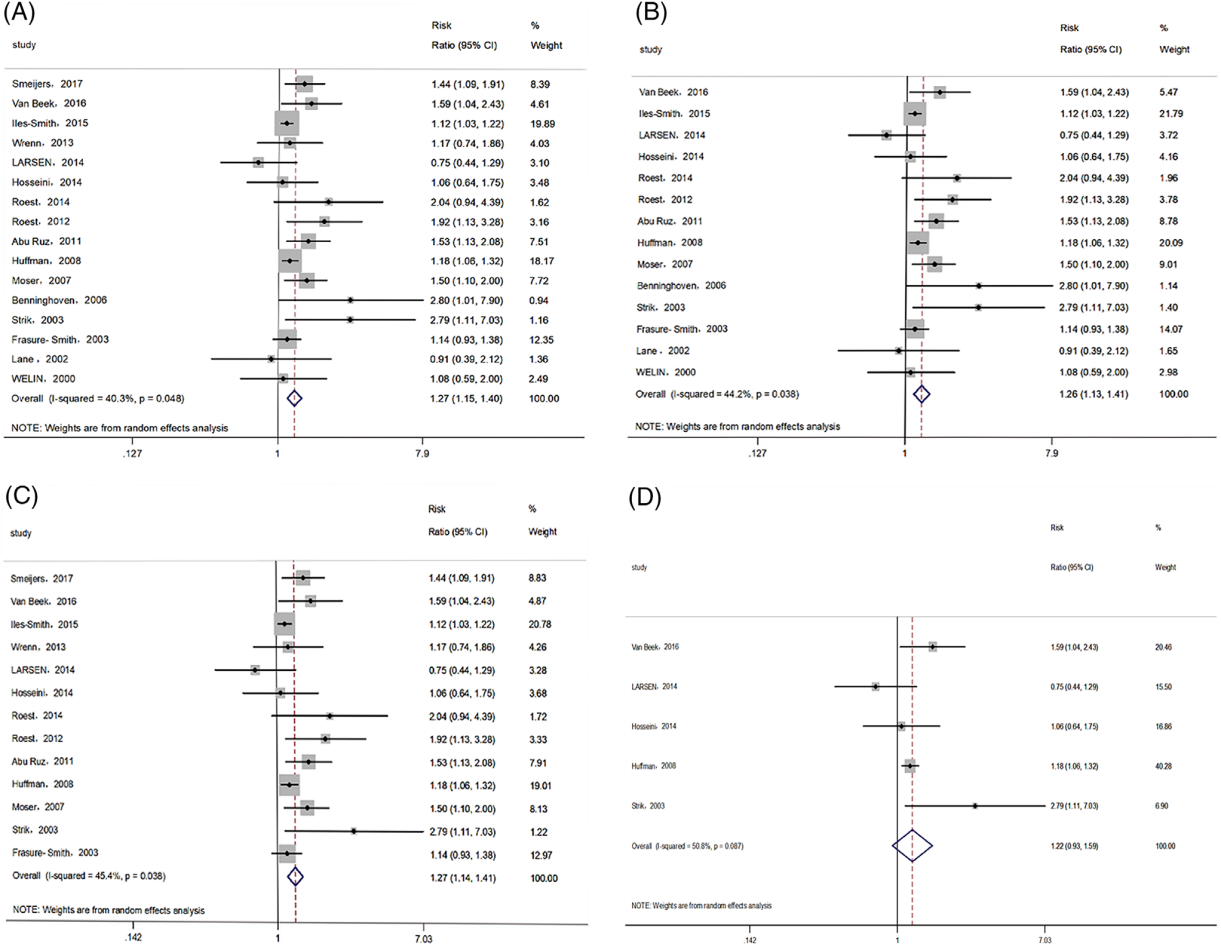
Forest plots of the relationship between anxiety and clinical outcomes in patients with MI (A), subgroup analysis of excluding studies in which anxiety were evaluated before MI (B), subgroup analysis of multivariable RRs (C), subgroup analysis after adjusting of depression (D). MI, myocardial infarction; RR, risk ratio

The 16 included studies reported a combined end‐point. We chose cardiac mortality as the end‐point if the study reported more than one end‐point. There was significant heterogeneity among the studies (I^2^ = 40.3%, P_h_ = 0.048); the pooled RRs for complications and all‐cause mortality and cardiac mortality and MACEs with corresponding 95% CIs were evaluated using a random‐effects model. The overall results demonstrated that MI patients with anxiety had a significantly poorer prognosis than those without anxiety (RR: 1.27, 95% CI: 1.15–1.40, *p* < .001; Figure 2(A)). Further analysis of excluding studies in which anxiety were evaluated before MI showed a consistent result (RR: 1.26, 95% CI: 1.13–1.41, *p* < .001; Figure 2(B)). The RRs for sensitivity analysis ranged from 1.25 to 1.31, which showed that the combined result was not significantly altered after excluding any studies. Subgroup analysis including 13 studies with multivariable RR also showed that anxiety was associated with poor prognosis in patients with MI (RR: 1.27, 95% CI: 1.14–1.41, P < 0.001; Figure 2(C)), but no significant association between anxiety and poor prognosis was observed after adjusting for depression (RR: 1.22, 95% CI: 0.93–1.59, *p* = .144; Figure 2(D)).

### 3.4 Anxiety and long‐term prognosis in patients with MI

**FIGURE 3:**
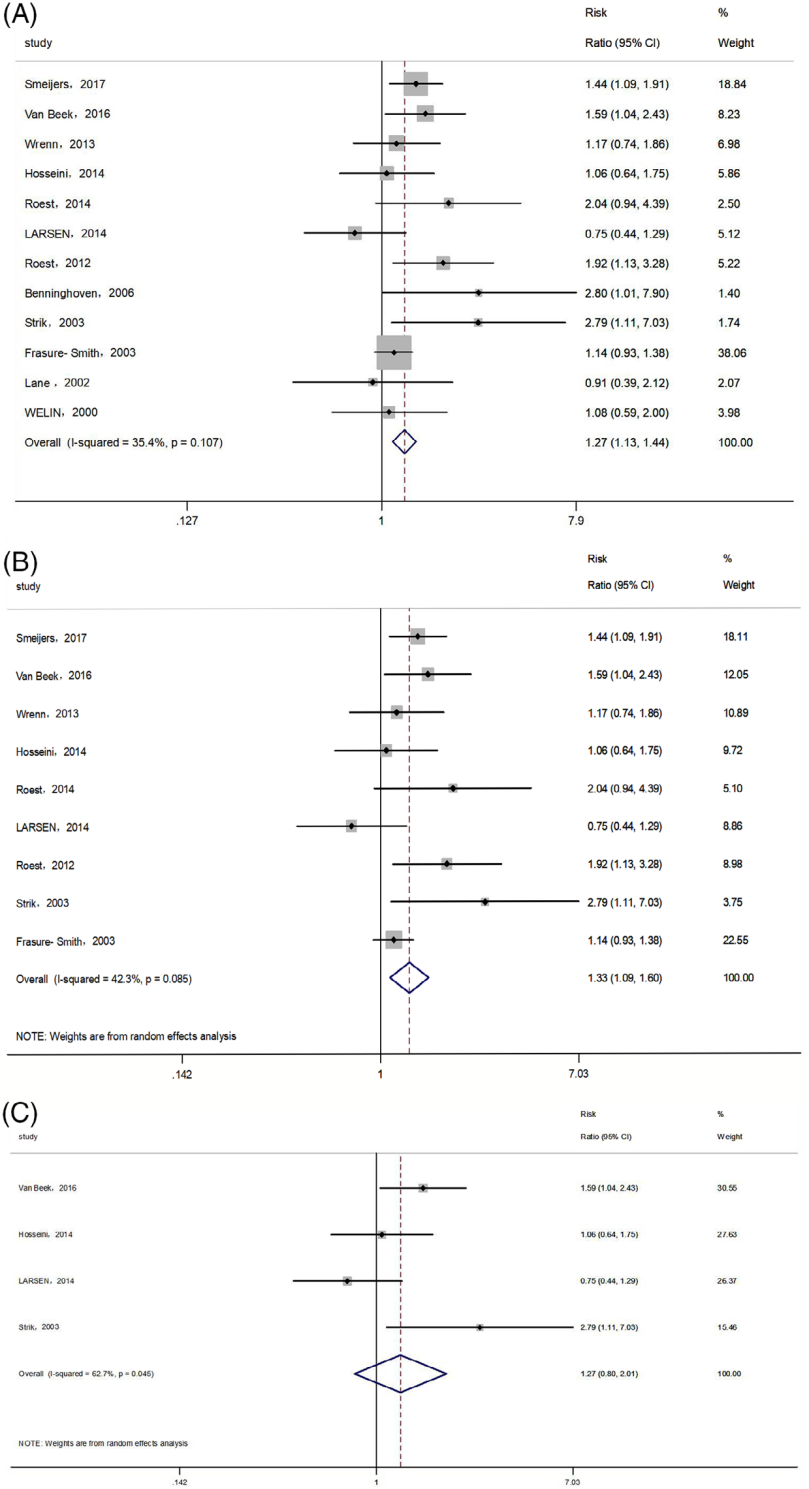
Forest plots of the relationship between anxiety and long‐term prognosis in patients with MI (A), subgroup analysis of multivariable RRs (B), subgroup analysis after adjusting of depression (C). MI, myocardial infarction; RR, risk ratio

Twelve studies, comprising 8204 patients with MI, reported the long‐term prognosis for all‐cause and cardiac mortality and MACEs. We chose cardiac mortality as the end‐point if the research reported more than one end‐point. The fixed‐effects model was used because there was no significant heterogeneity across the studies (I^2^ = 35.4%, P_h_ = 0.107). As shown in Figure 3(A), MI patients with anxiety had a worse prognosis than those without anxiety (RR: 1.27, 95% CI: 1.13–1.44, *p* < .001). The RRs for sensitivity analysis ranged from 1.27 to 1.36, which showed that the combined result was not significantly altered after excluding any studies. Subgroup analysis including nine studies with multivariable RR also showed that anxiety was associated with poor long‐term prognosis in patients with MI (RR: 1.33, 95% CI: 1.09–1.60, *p* = .004; Figure 3(B)), but we did not observe a significant association between anxiety and poor long‐term prognosis after adjusting for depression (RR: 1.27, 95% CI: 0.80–2.01, *p* = .306; Figure 3(C)).

### 3.5 Anxiety and long‐term mortality in patients with MI

Seven studies, including 6760 patients with MI, provided information on long‐term mortality analysis, which included all‐cause mortality and cardiac mortality. If the research reported both all‐cause mortality and cardiac mortality as endpoints, we chose cardiac mortality. The pooled results of the fixed‐effects model (I^2^ = 0.0%, P_h_ = 0.501) showed a significant association between anxiety and long‐term mortality (RR: 1.16, 95% CI: 1.01–1.33, *p* = .033; Suppl Figure 1A). The RRs for sensitivity analysis ranged from 1.08 to 1.19, which revealed that the combined result was not significantly altered after excluding any studies. The subgroup analysis including five studies with multivariable RR also showed that anxiety was associated with poor long‐term mortality in patients with MI (RR: 1.17, 95% CI: 1.02–1.35, *p* = .028; Suppl Figure 1B), but a significant association between anxiety and poor long‐term mortality was not observed in studies after adjusting for depression (RR: 0.90, 95% CI: 0.62–1.30, *p* = .575; Suppl Figure 1C).

### 3.6 Anxiety and long‐term all‐cause mortality in patients with MI

The association between anxiety and long‐term all‐cause mortality was reported in four studies including 5291 patients with MI. Significant heterogeneity was seen among studies (I^2^ = 50.1%, P_h_ = 0.111). The pooled results, which were evaluated by the random‐effects model did not show a significant association between anxiety and long‐term all‐cause mortality (RR: 1.14, 95% CI: 0.88–1.51, *p* = .355; Suppl Figure 2). The RRs for sensitivity analysis ranged from 1.01 to 1.27, which showed that the combined result was not significantly altered after excluding any studies.

### 3.7 Anxiety and long‐term cardiac mortality in patients with MI

Five studies comprising 3688 patients with MI reported the association between anxiety and long‐term cardiac mortality. Since no significantly heterogeneity was observed among studies (I^2^ = 0.0%, P_h_ = 0.985), a fixed‐effects model was used. The pooled results showed that there was no significant association between anxiety and long‐term cardiac mortality (RR: 1.12, 95% CI: 0.95–1.32, *p* = .165; Suppl Figure 3). The RRs for sensitivity analysis ranged from 1.08 to 1.13, which showed that the combined result was not significantly altered after excluding any studies.

### 3.8 Anxiety and long‐term MACEs in patients with MI

We evaluated the correlation between anxiety and long‐term MACEs in six studies including 2339 patients with MI. The pooled data using the fixed‐effects model (I^2^ = 24.2%, P_h_ = 0.252) indicated a significant association between anxiety and long‐term MACEs (RR: 1.54, 95% CI: 1.26–1.90, *p* < .001; Suppl Figure 4A). The RRs for sensitivity analysis ranged from 1.55 to 1.90, which showed that the combined result was not significantly altered after excluding any studies. The subgroup analysis including five studies with multivariable RR also showed that anxiety was associated with poor long‐term MACEs in patients with MI (RR: 1.51, 95% CI: 1.22–1.86, *p* < .001; Suppl Figure 4B), and a significant association between anxiety and poor long‐term MACEs was observed in studies after adjusting for depression (RR: 1.44, 95% CI: 1.14–1.82, *p* = .00; Suppl Figure 4C).

### 3.9 Anxiety and short‐term prognosis in patients with MI

Four studies, comprising 1170 MI patients, reported on short‐term prognosis. Significant heterogeneity was observed among the studies (I^2^ = 55.1%, P_h_ = 0.083), and a random‐effects model was used. The pooled results showed a significantly worse prognosis in MI patients with anxiety than those without anxiety(RR: 1.23, 95% CI: 1.09–1.38, *p* = .001; Suppl Figure 5). The RRs for sensitivity analysis ranged from 1.17 to 1.33, showing that the combined result was not significantly altered after excluding any studies.

### 3.10 Publication bias

**FIGURE 4:**
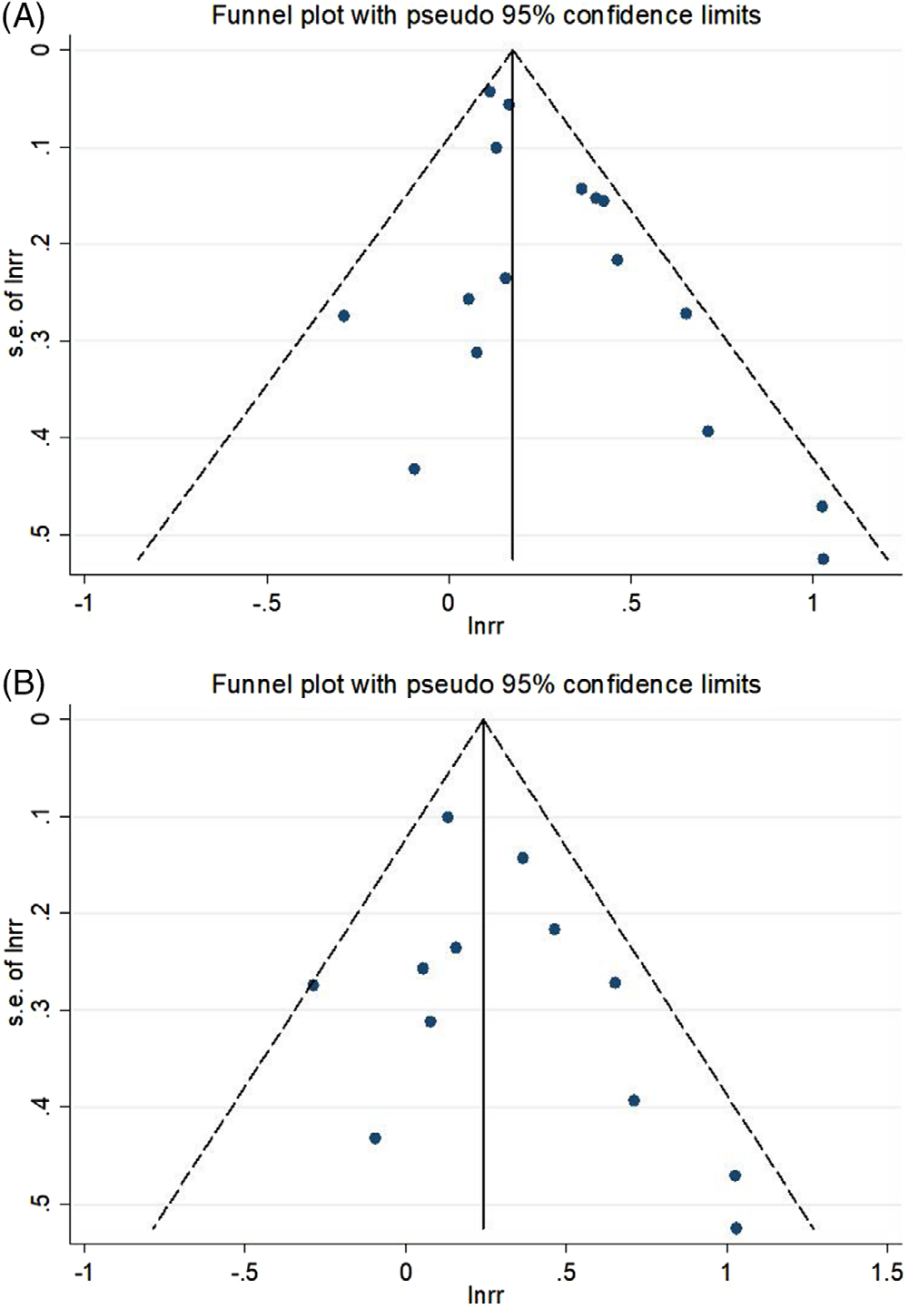
Funnel plots of the meta‐analysis of anxiety and prognosis in patients with MI. Clinical outcomes (A), long‐term prognosis (B). MI, myocardial infarction

For the meta‐analysis of the association between anxiety and combined clinical outcomes and long‐term prognosis and mortality, the funnel plots were symmetrical and are provided in Figure 4(A), (B).

## 4 DISCUSSION

MI has a high mortality, and patients with MI usually have a poor prognosis influenced by many factors, among which anxiety is an important factor. This meta‐analysis synthesized the prognosis of MI patients with or without anxiety and provided an updated estimate of the pooled RRs. We included 16 prospective studies comprising 9373 patients with MI. The prevalence of anxiety ranged from 5.5% to 58.2% in the eligible studies. We assessed short‐term and long‐term prognoses in MI patients with and without anxiety, including complications within 1 year, all‐cause mortality, cardiac mortality, and MACEs after 1 year. The results showed that MI patients with anxiety at baseline had a 27% greater risk of poorer clinical outcomes than those without anxiety. Further analysis revealed that MI patients with anxiety had a 23% increased risk of short‐term complications and a 27% increased risk of adverse long‐term prognosis compared to those without anxiety. This is consistent with data reported in the literature. Moser et al. found that anxiety during the in‐hospital phase of AMI was related to an increased risk for in‐hospital complications. Roest et al. found that anxiety is associated with poor long‐term prognosis in patients with MI (unadjusted 0R = 1.36). This meta‐analysis further evaluated the relationship between different long‐term clinical events. Anxiety was associated with a significantly increased risk of mortality or MACEs, with pooled RRs of 1.16 and 1.54, respectively. These results were consistent with those of a 2020 meta‐analysis by Li et al., in which the pooled RRs were 1.24 and 1.46 for mortality and MACEs, respectively. Furthermore, we found that anxiety was associated with a 1.14‐times higher long‐term all‐cause mortality rate and 1.12‐times higher long‐term cardiac mortality rate than those without anxiety.

The number of studies (*n* = 16) included in our meta‐analysis was greater than that in the abovementioned two previous meta‐analyses. There was no significant heterogeneity with I^2^ < 50%, except short‐term prognosis and long‐term all‐cause mortality. Sensitivity analysis of various outcomes showed that the combined result was not significantly altered after the exclusion of any studies. We further performed subgroup analyses including studies with multivariate RR for clinical outcomes, long‐term prognosis, long‐term mortality, long‐term MACEs; the RRs were 1.27, 1.33, 1.17, and 1.51, respectively. All our results were reliable. Previous studies have confirmed that both depression and anxiety were associated with poor prognosis in patients with MI.^14^, ^16^, ^23^ Some studies found that the association between anxiety and long‐term prognosis was insignificant after adjusted for depression.^23^, ^34^ And other studies shown the different results.^14^, ^16^ So in order to explore the potential influence of depression between anxiety and prognosis in patients with MI. We performed subgroup analyses after adjusting for depression; we observed no significant association between anxiety and poor clinical outcomes, poor long‐term prognosis, poor long‐term mortality (*p* > .05). However, there was a significant association between anxiety and poor long‐term MACEs (*p* = .002). The results suggest that depression may be a contributing factor for the association between anxiety and prognosis in patients with MI. However, we included only five studies in the subgroup analysis; hence, the results may be unreliable, and further analyses should be performed with a larger sample size.

In two previous meta‐analyses, MI patients with anxiety had a poorer prognosis than those without anxiety.^34^, ^35^ In one meta analysis, patients with MI and unstable angina were included, the results were assessed using bivariate analysis, and there was a significant publication bias in the combined outcomes.^35^ The other meta‐analysis evaluated the effect of anxiety in patients with ACS, and included patients with depression or other factors.^34^ In addition, both the meta‐analyses focused on only long‐term prognosis, and did not analyze the association between anxiety and the short‐term prognosis of patients with MI. Therefore, we performed an updated meta‐analysis to compare the clinical outcomes in MI patients with and without anxiety. Compared to the aforementioned two previous meta‐analyses, our updated meta‐analysis has three strengths. First, we only included MI patients, with no other pathology like unstable angina. Second, we only considered anxiety as the exposure factor, and studies that considered other exposure factors, such as depression or perceived control, were excluded. Third, in addition to long‐term prognosis, we also paid attention to short‐term prognosis. Furthermore, various clinical outcomes were evaluated in MI patients with and without anxiety. Overall, our meta‐analysis provided more reliable results than the aforementioned studies and reinforced previous findings that showed that anxiety is associated with a mild risk for poor prognosis.

The pathophysiological mechanism underlying the adverse association between anxiety and cardiac prognosis is complicated and unclear.^36^ Some hypotheses, such as neuro‐humoral mechanisms, platelet activation, endothelial dysfunction, and multi‐organ network, may help explain this association. Because of decreased vagal tone, anxiety is related to reduced baroreflex control of the heart and increased arrhythmia in patients with AMI.^37^ Anxiety is also potentially related to hypothalamic–pituitary–adrenal axis dysregulation.^38^ Platelet aggregation is a main contributor to the development of MI. Anxiety is associated with higher platelet reactivity, thus enhancing platelet aggregation and leading to clot formation.^39^, ^40^ Anxiety is associated with poor endothelial and vascular smooth muscle function in patients with atherosclerosis and has been shown to increase vascular events in patients with MI.^41^ Recently study have highlighted the multi‐organ network linking brain emotional neural activity, macrophage hematopoiesis, and arterial inflammation of AMI. Brain emotional neural activity is closely linked with acute plaque instability through enhanced hematopoietic macrophage activity.^42^

This updated meta‐analysis has some limitations. First, the number of included studies was relatively small, and the further studies with a larger sample size is needed. Second, anxiety was evaluated using nine different instruments in the included studies, with no agreed standard to judge whether the patient had anxiety. The timing of evaluation was different in the 16 studies; two studies evaluated anxiety prior to MI based on patients' memories.^15^, ^26^ Third, the follow‐up duration was variable in the included studies, ranging from 1.3 to 10 years. Fourth, publication bias may exist; however, no significant publication bias was observed based on the funnel plot and the sensitivity analysis showed stable results. Overall, all these limitations may have affected the results.

In conclusion, our study provided strong evidence that increased anxiety was associated with poor prognosis in patients with MI. Well‐designed clinical studies including a larger sample size and other ethnic groups are needed to further confirm the association between anxiety and prognosis in patients with MI, to determine the optimal management methods for MI patients with anxiety, which can improve their prognosis.

## CONFLICT OF INTEREST

The author declare no potential conflict of interest.

## Supporting information


**Appendix** S1: Supporting Information

## Data Availability

The data supporting this meta‐analysis are from previously reported studies and datasets, which have been cited.
