# Supplementary material for: Anxiety and prognosis of patients with myocardial infarction: A meta‐analysis
Source: Clin Cardiol. 2021 May 7;44(6):761–70. doi: 10.1002/clc.23605 (PMC8207975; doi:10.1002/clc.23605)
Supplement: Supplementary file 1 — Appendix S1: Supporting Information [file CLC-44-761-s001.docx]

**Supplementary material**

**Suppl Table 1. Quality assessment of cohort studies.**

| study | selection | | | | comparability | outcome | | | total |
| --- | --- | --- | --- | --- | --- | --- | --- | --- | --- |
|  | 1 | 2 | 3 | 4 | 1 | 1 | 2 | 3 |  |
| Smeijers,2017 |  | ☆ | ☆ | ☆ | ☆ | ☆ | ☆ | ☆ | 7☆ |
| Van Beek,2016 |  | ☆ |  | ☆ | ☆ | ☆ | ☆ | ☆ | 6☆ |
| Iles-Smith,2015 | ☆ | ☆ |  | ☆ | ☆ | ☆ |  | ☆ | 6☆ |
| LARSEN,2014 |  | ☆ |  | ☆ | ☆ | ☆ | ☆ | ☆ | 6☆ |
| Hosseini,2014 | ☆ | ☆ |  | ☆ | ☆ | ☆ | ☆ | ☆ | 7☆ |
| Roest,2014 | ☆ | ☆ |  | ☆ | ☆ | ☆ | ☆ | ☆ | 7☆ |
| Wrenn,2013 | ☆ | ☆ | ☆ | ☆ | ☆ | ☆ | ☆ | ☆ | 8☆ |
| Roest,2012 |  | ☆ | ☆ | ☆ | ☆ | ☆ | ☆ | ☆ | 7☆ |
| AbuRuz,2011 |  | ☆ | ☆ | ☆ | ☆ | ☆ |  | ☆ | 6☆ |
| Huffman,2008 |  | ☆ | ☆ | ☆ | ☆ | ☆ |  | ☆ | 6☆ |
| Moser,2007 | ☆ | ☆ | ☆ | ☆ | ☆ | ☆ |  | ☆ | 7☆ |
| Benninghoven,2006 |  | ☆ | ☆ | ☆ |  | ☆ | ☆ |  | 5☆ |
| Strik,2003 |  | ☆ |  | ☆ | ☆ | ☆ | ☆ | ☆ | 6☆ |
| Frasure-Smith,2003 | ☆ | ☆ |  | ☆ | ☆ | ☆ | ☆ | ☆ | 7☆ |
| Lane,2002 | ☆ | ☆ |  | ☆ |  | ☆ | ☆ | ☆ | 6☆ |
| WELIN,2000 | ☆ | ☆ |  | ☆ |  | ☆ | ☆ | ☆ | 6☆ |

**Figure legend**

Suppl Figure 1. Forest plots of the relationship between anxiety and long-term mortality in patients with MI(A), subgroup analysis of multivariable RRs(B), subgroup analysis after adjusting of depression(C). MI, myocardial infarction. RR, Risk Ratio.

Suppl Figure 2. Forest plot of the relationship between anxiety and long-term all-cause mortality in patients with MI. MI, myocardial infarction.

Suppl Figure 3. Forest plot of the relationship between anxiety and long-term cardiac mortality in patients with MI. MI, myocardial infarction.

Suppl Figure 4. Forest plots of the relationship between anxiety and long-term MACEs in patients with MI(A), subgroup analysis of multivariable RRs(B), subgroup analysis after adjusting of depression(C). MI, myocardial infarction. RR, Risk Ratio.

Suppl Figure 5. Forest plot of the relationship between anxiety and short-term prognosis in patients with MI. MI, myocardial infarction.

Suppl Figure 1


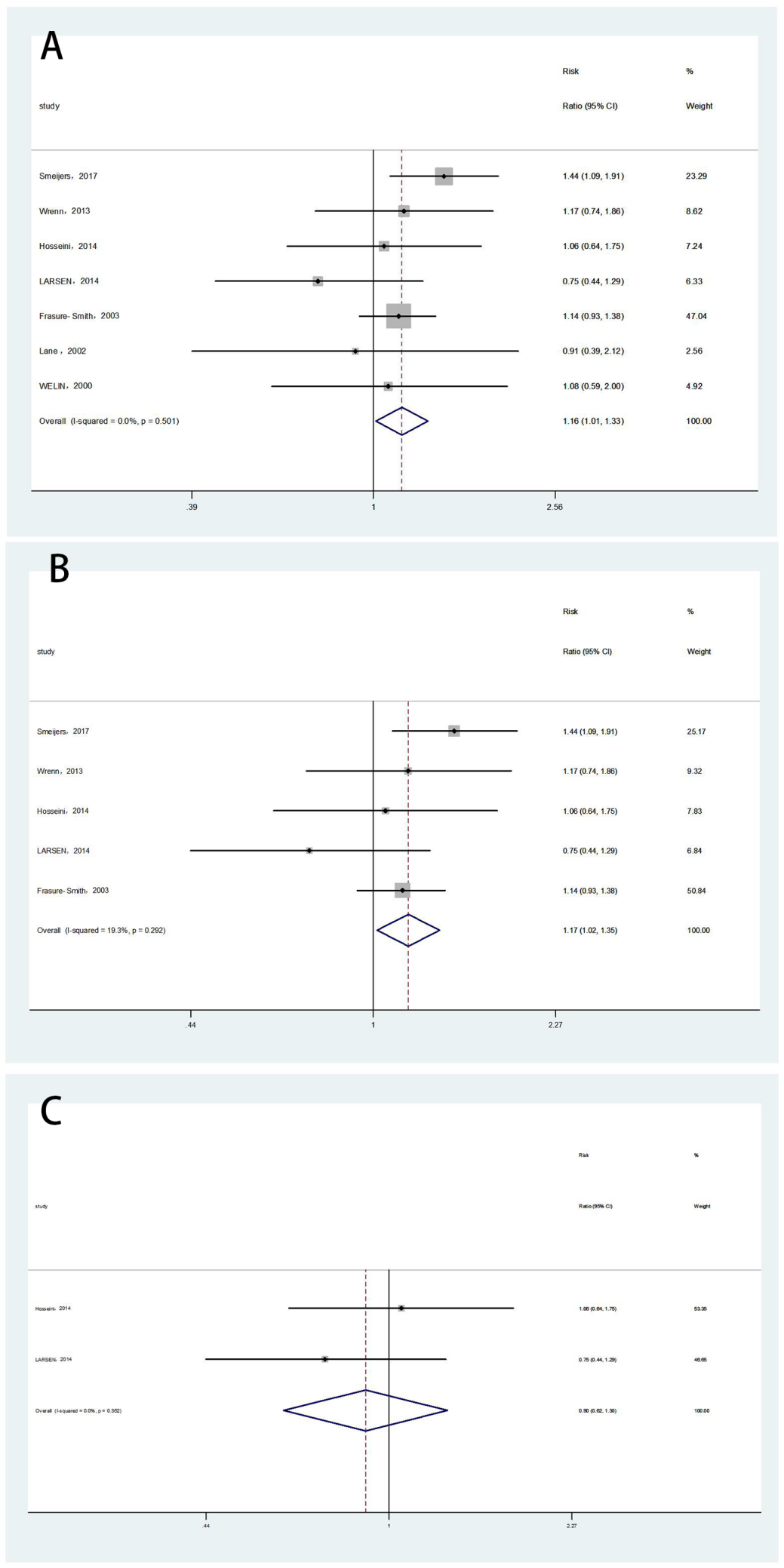


Suppl Figure 2


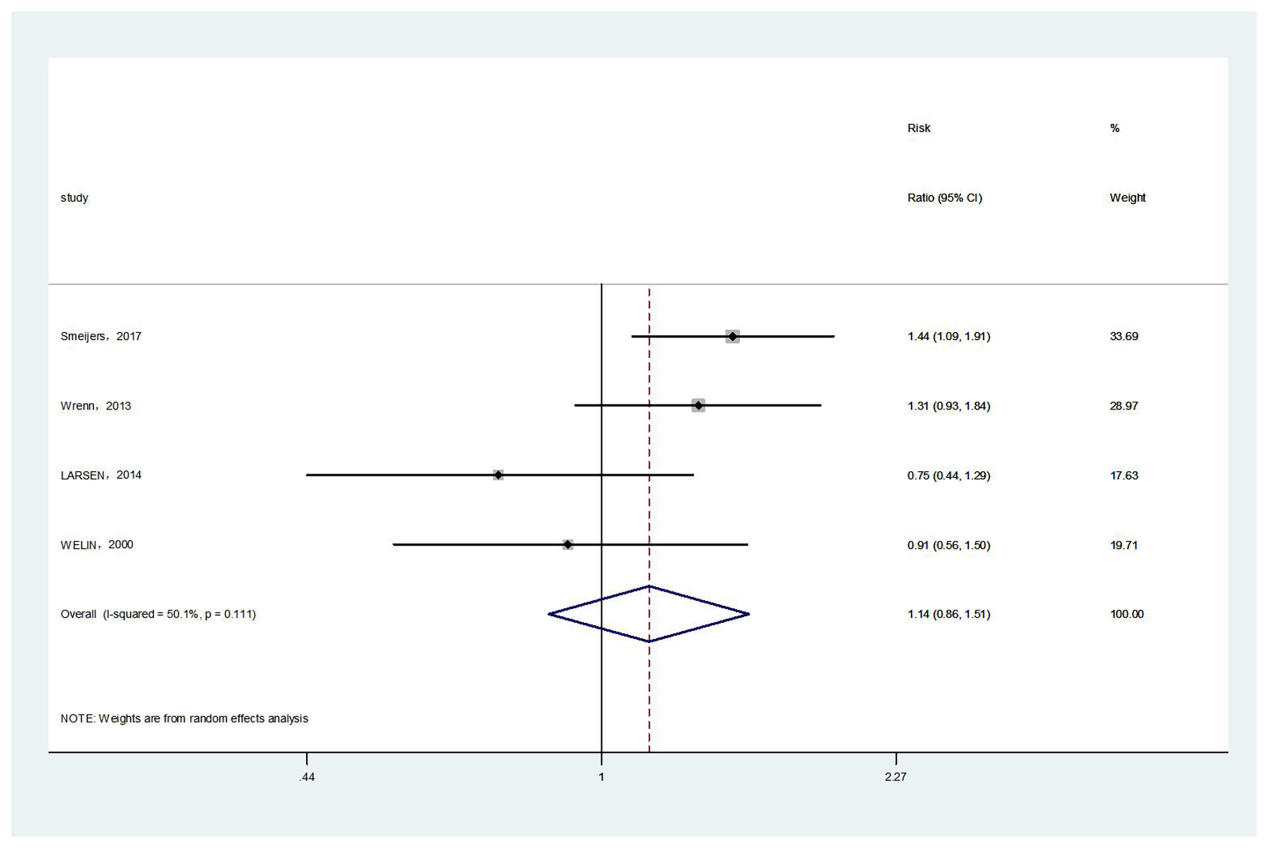


Suppl Figure 3


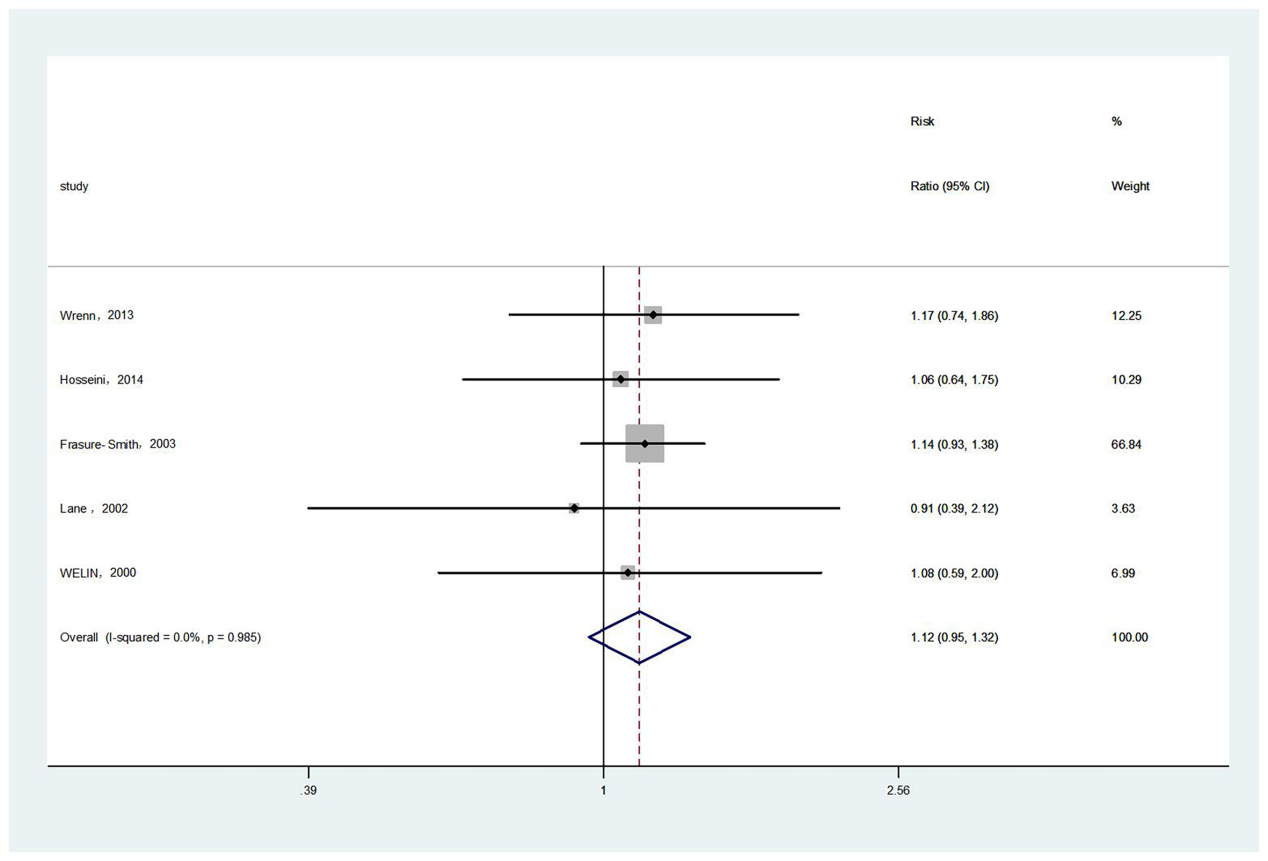


Suppl Figure 4


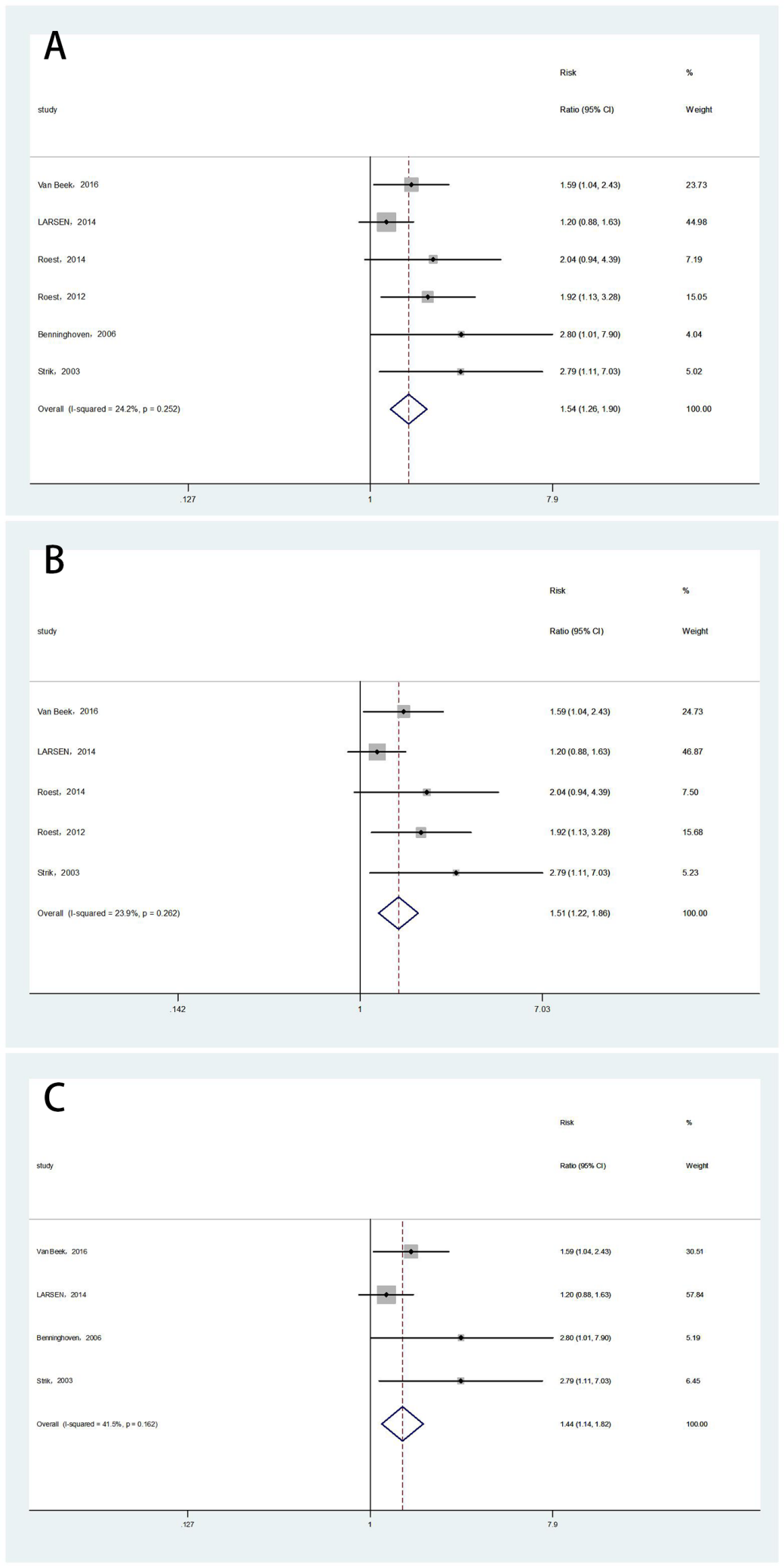


Suppl Figure 5

**
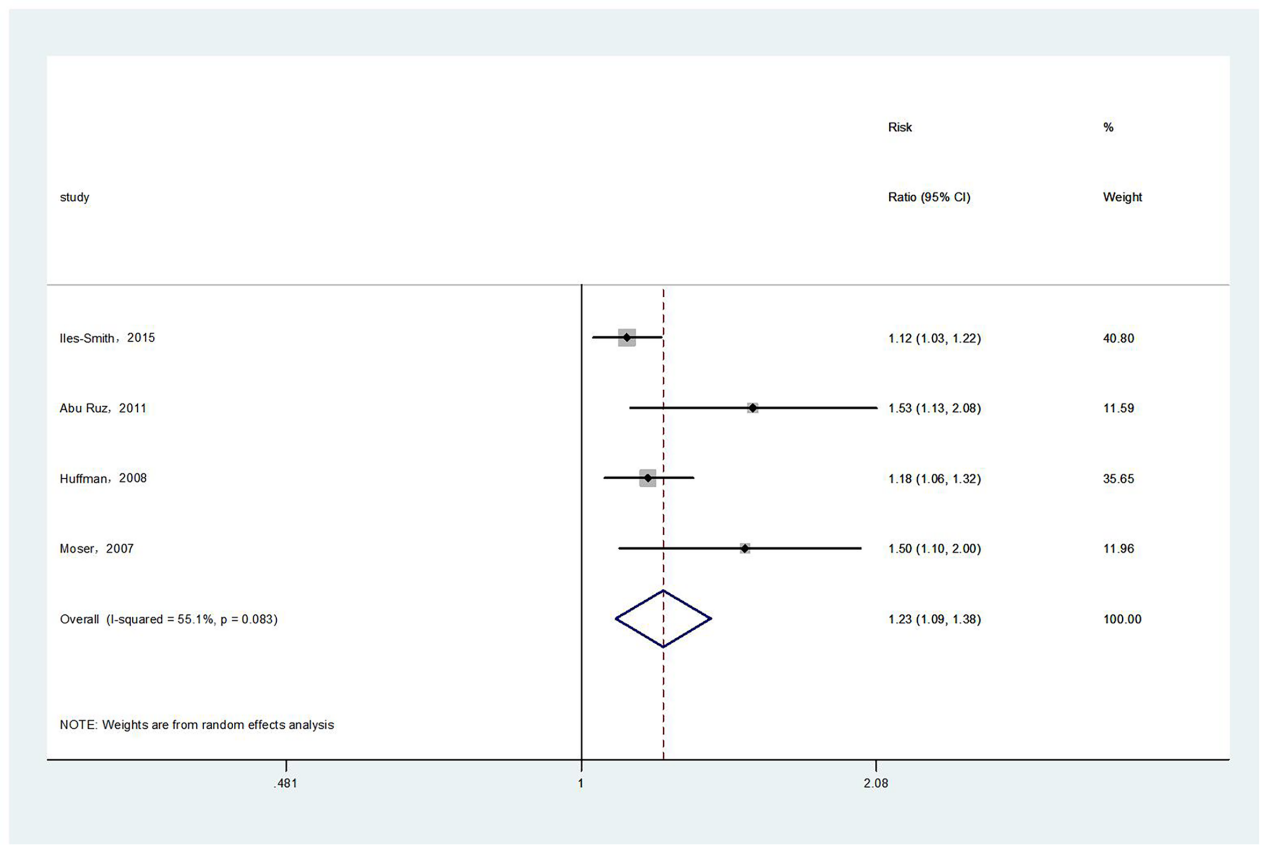
**
